# Hidden populations: discovering the differences between the known and the unknown drug using populations in the Republic of Georgia

**DOI:** 10.1186/s12954-019-0287-5

**Published:** 2019-02-12

**Authors:** M. Gogia, C. Lawlor, N. Shengelia, K. Stvilia, H. F. Raymond

**Affiliations:** 1 The Georgian Harm Reduction Network, Tbilisi, Georgia; 2 Curatio International Foundation, Tbilisi, Georgia; 3 The National Centre for Disease Control and Public Health, Tbilisi, Georgia; 4 School of Public Health, Rutgers University, New Brunswick, NJ USA

**Keywords:** PWID, Georgia, HIV, Knowledge, Behaviours, Peer-Driven Intervention, PDI, Needle and syringe program, NSP

## Abstract

**Background:**

The HIV epidemic in Georgia is increasing. Data shows that compared to previous years, Georgia has increasingly more HIV-infected individuals than previous assessments. Select client groups remain hard to reach by harm reduction programs. The need for innovative strategies to involve these individuals is imperative.

**Methods:**

The following study examines demographics and risk factors of participants, previously known and not known to harm reduction services, for HIV and other infectious disease in towns across Georgia in 2015 and compares risk among different groups, while also assessing the rationale for implementing Peer-Driven Interventions in Georgian Harm Reduction activities. Important differences in demographics and risk profile are thought to exist between those exposed, and those unexposed, to harm reduction activity.

**Results:**

Important and striking differences between previously known and unknown participants, including demographic background and risk profile and behaviours exist in the drug using community. These differences can potentially explain some of the rise of HIV prevalence in Georgia.

**Conclusion:**

Significant differences exist between known and unknown drug users in Georgia, the differences between which are crucial for planning future and holistic harm reduction activities in Georgia, regionally and globally. The research advocates for smarter harm reduction activity, adds to the global evidence for the utility of Peer-Driven Intervention, and encourages sustained global effort for reduction of blood-borne disease burden globally.

## Introduction

Despite low HIV prevalence in the general population (0.07%), Georgia faces a considerable risk of an expanding HIV epidemic due to widespread high-risk practices among key affected populations; people who inject drugs (PWIDs) and men who have sex with men (MSM) [1], and needle-sharing practices have remained a significant factor for spreading HIV among people who inject drugs (43.8%) [1]. Moreover, there is a significant risk of sexual transmission of HIV to the general public through bridging populations [2]. Trends suggest a growing spread of HIV among the sexual partners of PWIDs [3].

The estimated population size of injecting drug users (PWIDs) has increased during recent years in Georgia and in 2015 was estimated at 49,700, 1.41% of the 3.5 million Georgians [4]. Needle-syringe programs (NSPs) have been active in the country since 2005, but on a limited scale. The Georgian Harm Reduction Network (GHRN) together with 10 NGOs is implementing the Global Fund-funded NSP program with 14 harm reduction sites in 11 cities of Georgia. The community-based harm reduction services are now reaching between 10,500 and 12,000 PWIDs per month, three times more than in 2011.

Needle and syringe programs provide a basic package of services to PWID, including distribution of sterile injection equipment, voluntary counselling and testing (VCT) for HIV, HCV, HBV and syphilis, distribution of safe sex information and prophylactics, and overdose prevention materials, such as naloxone**.**

NSP prevention efforts targeting PWIDs resulted in positive injecting behaviour change, with 78.4% and 89% of PWIDs using sterile injecting equipment in the cities of Batumi and Tbilisi respectively, showing progressive improvement since 2009, according to the last Integrated Bio-Behavioural Surveillance Survey (IBBSS) [5]. The number of needles and syringes distributed per PWID per year by NSP in Georgia has increased (25.2 in 2012 to 80.3 in 2015) [6]; although, it remains low compared to WHO recommended level of 200 per injector per year [7]. Continued criminalisation of drug consumption is likely a reason for underground PWID activity and results in increased use of home-made drugs [8] that are mostly used in groups and several times per day [5].

In order to increase HIV testing coverage and attract a greater segment of PWID, for example female and young injectors, Peer-Driven Intervention (PDI) have been introduced in the NSP program in Georgia since 2009. Peer-Driven Intervention is a system whereby PWID are trained to recruit and disseminate information into their drug network, as to provide harm-reducing education and as an introduction to harm reduction services. Based on a multi-year field experiment, PDI is effective in guiding HIV educational intervention. Besides recruitment and education, PDI can allow for collection of information, such as social demographics, HIV testing practice, injecting and sexual behaviour, and HIV knowledge, among PWIDs that have no experience with harm reduction programs. After involvement in PDI, each recruited drug user can receive sterile injecting equipment, naloxone, condoms, informational-educational materials, medical consultations, and supplementary services. Based on this sampling, PDI-involved drug users can invite PWIDs from their acquaintance network.

Results of PDIs in other locations show that they are effective in recruiting younger PWIDs and can reduce injection frequency, reduce rates of sharing of syringes and equipment, and reduce rates of unprotected sex [9]. Research shows that PDI populations have statistically significant differences from NSP participants in terms of knowledge and risk taking [10]. Compared with the traditional model, PDI reaches a larger and more diverse group of PWID and does so at substantially lower expense [10]. By using PDIs, NSP programs have gained access to a high-risk group with important differences in knowledge and risk who had remained unknown to harm minimisation programs.

We present the findings of a pilot PDI program in Georgia, with the aim to build on preliminary global research and to quantify and compare the differences in demographic information and risk profile of PWIDs previously known to NSP program and those who were previously unknown. The aim of this is to discover important differences as to why PWIDs remain unknown to harm reduction services; to plan more inclusive, holistic services; and to contribute to the evidence base for use of PDI in harm minimisation programs in Georgia that could be applied to other regional locations with similar societal demands, especially post-Soviet nations, and global contexts. It is hypothesised that important demographic and risk differences currently exist between PWIDs known to harm reduction activities and those who remain hidden populations to these activities. We show the main demographic differences between the two groups (current clients and PDS-recruited PDIs), compare the risk behaviours of two population groups, and propose whether NSP participation results in risk behaviour change by comparing the differences in program exposure.

## Methods

This cross-sectional study was conducted among PWIDs in 2015. Study participants were recruited in 13 harm reduction sites over 8 months (January to September). Two sampling strategies were applied. The inclusion criteria for both study populations were (1) drug injection during last month, (2) age of 18 or over, and (3) willingness to participate voluntarily in the study. Additional inclusion criteria included being a beneficiary of NSP program for more than 6 months for the NSP sample and for PDI sample, not having been engaged in any HIV prevention program prior to being recruited into the study.

First, consecutive sampling was used for NSP program participants. All NSP clients who agreed to participate and met the pre-established selection criteria were involved in the study until the required sample size was reached. Before the initiation of NSP study, information about the study was spread among program participants: the study announcement was available on all service centre walls, and outreach workers offered their clients to take part in the study. The announcement included brief information about the purpose and importance of the study. Study staff were not involved in selecting participants for NSP study.

Second, Respondent-driven sampling (RDS) was used to recruit PWID from the community. The recruitment started with non-randomly selected PWIDs, called “seeds”, which were prepared and familiarised with study objectives. At each study site 4–6 seeds were selected by VCT consultants from diverse groups (gender, age, injecting drug type, years of drug injection, location). The seeds, and all afterwards recruited PWIDs, who met the study inclusion criteria, were given three coupons to recruit three new PWIDs in the study. The chain continued until the sample size was achieved. The coupons were the main tools for RDS sampling, giving the possibility to recruit new PWIDs from the seed’s network, only among his/her acquaintances. Each coupon’s unique code included information to track which PWIDs were recruited by whom. No PWIDs were included in the study without coupons. Information was recorded in a coupon management database that automatically generated coupon numbers for each delivered coupon. Each participant could take part in PDI only once.

Interviewer-administered face-to-face questionnaires were performed to study basic socio-demographic and behavioural characteristics of PWIDs in both samples in 10 cities of Georgia (Tbilisi, Rustavi, Telavi, Gori, Zugdidi, Poti, Batumi, Ozurgeti, Kutaisi, and Samtredia). The study interviewers were trained based on study protocol, interviewing techniques, and instruments. The main part of the questionnaire included a modified Risk Assessment Battery (RAB) [11] to assess drug risk and sex risk behaviour separately and calculate a total RAB Score (drug risk total + sex risk total) for these populations. All the conducted interviews were anonymous. PWIDs that were recruited by RDS were interviewed to assess previous PDI involvement to avoid any influence of these interventions in their answers to the questions posed.

Each PDI study participant received incentives for study participation, recruitment, and education of peers. Incentives for education were received by educators: PWIDs that were recruited and educated by PDI. The amount of reimbursement was dependent on the number of correct answers on eight questions done by their recruited PWIDs. Additional incentives were given for recruitment of young PWIDs (under 25 years) and female PWIDs. All study participants signed two written consent forms for study participation and recruitment and education. Based on implemented tasks, minimum reward for PDI study participants was 10 Georgian Lari (GEL) (4 USD) and maximum 50 GEL (20 USD), with an average of 30 GEL (13 USD). Additionally, all participants were offered free testing for HIV, HBV/HCV and syphilis, and other services from a comprehensive harm reduction package. No incentives, besides program services, were given to NSP study participants. The data was collected by 23 experienced interviewers and entered in electronic databases in Dropbox. The quality of data entry was supervised online by the principal investigator.

To test the study hypothesis, it was assumed that PWIDs with more than 6 months NSP program experience would have familiarity with harm reduction programs, and their knowledge and risk characteristics could be compared to PDI-recruited PWIDs who had no previous access to harm reduction programs and the education that comes with involvement.

## Analysis

We calculated descriptive statistics for both study samples. We dropped missing data including “hard to answer” and “refuse to answer” responses before performing further analysis. In bivariate analysis, we compared categorical variables by chi-square test. We examined relationships between age groups and risky sexual and injecting behaviour in separate and in total, as well between gender and risky sexual and injecting behaviour. SPSS V.22 was used for the analysis.

Risky behaviour was assessed by Risk Assessment Battery (RAB) Scoring System [11]. According to the RAB methodology, 16 items from the RAB are used in the computation of three scores: a drug-risk score, a sex-risk score, and a total score. The eight-item drug-risk score has a range of 0 to 22. The range of the sex-risk score, comprised of nine items, is 0 to 18. This simple scoring system was designed to capture frequency of engaging in each of the reported risk behaviours. Scores for the various items are not differentially weighted. This scoring strategy serves to guard against underestimates of risk resulting from the tendency to under report participation in behaviours known to be most likely to transmit the HIV virus [11]. Risk assessment score and index were calculated within each sample. We have derived the raw mean score out of the respective “drug” and “sex risk” questions and a mean score for that group (Table 1—simplified drug and sex risk, Table 3—full results). Descriptive statistics were calculated for the both study samples, including the 95% confidence interval around the mean and a *p* value for each of the comparative risk sections (Table 3). Finally, to look at the statistically significant differences within the groups, we used one-way ANOVA tests for both PDI and NSP samples separately.

**Table 1:** Risk Assessment score and Index by sample type

Sample type	Drug risk	Sex risk	Total risk
PDI *N* = 1939			
Mean score	7.17	12.17	19.34
Mean index	0.33	0.68	0.48
NSP *N* = 1032			
Mean score	11.81	14.85	26.67
Mean index	0.54	0.83	0.67

## Results

In total, 1032 NSP participants and 1939 PDI participants were interviewed during the study (Table 2). Significant numbers of PWIDs were recruited from the capital of the country (Tbilisi) in both the NSP (24.8%) and PDI (38.4%) sample. Mostly heterosexuals and men took part in the study; the portion of women was not substantial in both samples (11.5% in NSP and 9.3% in PDI). PDI sampling enabled recruitment of younger (18–25) PWIDs (17.7% of the PDI group); while this age group in the NSP study sample was small (4.5%). PWIDs in both samples had the same education level, were mostly unemployed (NSP 47.0% and PDI 60.1%), and a small number lived in their own housing (NSP 39.7% and PDI 28.6%).

**Table 2:** Characteristics of sample in 10 cities of Georgia, 2015

Characteristics	NSP *N* = 1032 %	PDI *N* = 1939 %	*p* values
Demographics			
Age			
= < 25	4.5	17.7	*0.00*
= > 26	95.5	82.3	
Gender			
Male	88.5	90.7	*0.05*
Female	11.5	9.3	
Education			
Incomplete or complete secondary	43.2	46.9	0.12
Higher education, complete or incomplete	42.3	38.6	
College or professional institution/ or incomplete	14.5	14.5	
City of residence			
Tbilisi	24.8	38.4	*0.00*
Rustavi	7.3	9.9	
Gori	6.8	4.7	
Batumi	6.8	7.1	
Ozurgeti	14.5	8.8	
Kutaisi	6.8	8.5	
Samtredia	5.7	7.8	
Poti	13.6	7.3	
Zugdidi	7.9	5.9	
Telavi	5.8	1.5	
Employment			
Unemployed	47.0	60.1	*0.00*
Employed	53.0	39.9	
Place of residence			
Own housing	39.7	28.6	*0.00*
Parents/relative/friends housing	50.6	58.1	
Rent/Mortgage/shelter/homeless	9.7	13.3	
Drug use behaviour			
Age at first injecting drug			
Mean	20.5	19.6	*0.00*
Years of injection			
Mean	14.8	12.7	*0.00*
Frequency of Drug injection during last 30 days			
Mean	9.12	9.3	0.55
Safe Injection practice during the last 6 months			
Never shared Injection equipment or syringes	49.2	34.1	*0.00*
Never using syringes previously used by others during the last 6 months			
Never shared	65.5	44.7	*0.00*
Never giving others previously used syringes by him/herself during the last 6 months			
Never shared	62.6	44.2	*0.00*
Sharing practice with someone they knew had AIDS during the last 6 months			
Never	96.1	96.6	*0.03*
Few times or less	3.4	2.2	
Few times each month	0.3	0.3	
Once or more each week	0	0.7	
No response	0.2	0.3	
More usable (injected) drugs			
Desomorphine (crocodile)	27.7	16.1	*0.00*
Heroin, sirets	45.4	53.1	*0.00*
Street methadone	7.1	6.9	*0.00*
Suboxone/Subutex	24.7	29.2	*0.00*
Vint, jeff	17.7	24.0	*0.00*
Tropicamid, Ketamine, Calypso	7.8	9.1	*0.00*
Antihistamines in mixture	5.6	4.0	*0.00*
How did they survive their last overdose?			
Ambulance	2.8	5.6	*0.37*
Salty water injection	3.1	8.2	
Naloxone injection	13.4	6.1	
Sex behaviour			
Sexual orientation identity			
Heterosexual	99.7	97.6	*0.00*
Ever exchange of sex for drugs			
Yes	3.3	5.9	*0.00*
Ever exchange of sex for money			
Yes	16.4	20.2	*0.01*
Condom use during the last 6 months			
Did not have sex during the last 6 months	23.5	20.2	*0.00*
Always	27.7	17.7	
Mostly	31.0	39.2	
Sometimes	14.1	17.2	
Never	0.1	0	
No response	3.5	5.6	
HIV testing practice and knowledge			
Tested on HIV			
Never tested	20.4	70.2	*0.00*
Knows HIV status^*^			
Yes	97.9	89.8	*0.00*
HIV testing period^*^			
During the last month	9.5	3.5	*0.00*
During the last 6 months	49.1	18.4	
During more than 1 year	40.6	76.9	
No response	0.9	1.2	
HIV knowledge^▪^			
Yes	75.2	53.0	*0.00*

^*^Among those, who were tested

^▪^Correctly answers on all 5 questions (Does your chance of infection decrease if your sexual partner is not infected and does not have sex with others? Is there a chance to reduce HIV infection risk if a person uses a condom every time during sex? Do you think that a healthy looking person may be HIV-positive? Can a person get HIV by using food or water of an HIV-infected person? Can HIV infection be transmitted by mosquito bite?) about HIV transmission

Age at first injection of a drug was the similar in the both samples (NSP median 20.5 and PDI median 19.6). The NSP sample PWIDs had more injecting experience (median 14.8 years) than the PDI participants (median 12.7 years). NSP study participants reported having a higher knowledge about naloxone and safer survival measures during an overdose. Naloxone use rate, expressed as whether naloxone was used during participants’ last overdose, was 13.4% in the NSP sample compared to 6.1% in the PDI sample.

**Fig. 1:**
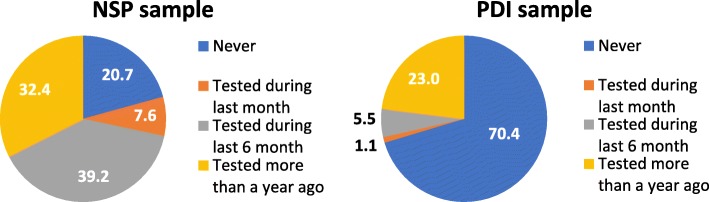
HIV testing practice among PDI and NSP participants

Lifetime HIV testing practice was low among PDI participants with 70.2% never tested and 23.1% tested more than a year prior. HIV testing frequency was also low among PDI participants; most were tested once (20.2%) and less were tested twice (4.9%) and three times or more (3.8%). NSP participants reported higher HIV testing frequency, with 20.7% never tested, 39.2% tested during the last 6 months, and 32.4% tested more than a year prior. Besides these, more NSP participants were tested more than once during their lifetime, with 29.6% tested once, 21.3% tested twice, and 28.5% tested three times or more (Fig. 1). These figures could be expected, due to the interaction with harm reduction services, which offer testing during client interaction.

Risk Assessment Battery (RAB) Scoring System results showed that drug use and sexual behaviour risks were different among NSP participants in terms of raw figures; their risky behaviour is more related to unsafe sex (Sex Index—0.83) than unsafe drug injecting behaviour (Drug Index—0.54) (Tables 1 and 3). The same situation was revealed in PDI sample; sexual behaviour is riskier than injecting behaviour (0.68 v. 0.33). The differences in risk between the groups have been further analysed in Tables 2 and 3, and *p* values derived to show statistically significant differences. In the NSP sample, age under 25 was statistically insignificant for overall greater risk behaviour (0.68 v. 0.67, *p* value = 0.45), whereas in the PDI sample, age over 26 was significant for greater drug (0.33 v. 0.31, *p* value = 0.02), sex (0.69 v. 0.62, *p* value = 0.00), and overall risk (0.49 v. 0.45, *p* value = 0.00). Women undertook less risky behaviours in both samples, which was statistically significant for all risk categories, except overall risk in the NSP group (overall risk 0.64 v. 0.67, *p* value = 0.09). It is unknown why these differences exist between these groups in Georgia.

**Table 3:** Comparison of Risk Assessment Index within some groups

Sample characteristics		Sample type											
		NSP						PDI					
	Groups	Drug index 95% CI	*p* values	Sex index 95% CI	*p* values	Risk index 95% CI	*p* values	Drug index 95% CI	*p* values	Sex index 95% CI	*p* values	Risk index 95% CI	*p* values
Age	= < 25	0.56 (0.47:0.65)	0.54	0.84 (0.76:0.91)	0.77	0.68 (0.64:0.73)	0.45	0.31 (0.29:0.32)	*0.02*	0.62 (0.6:0.65)	*0.00*	0.45 (0.43:0.46)	*0.00*
	= > 26	0.54 (0.52:0.55)		0.82 (0.81:0.84)		0.67 (0.66:0.68)		0.33 (0.32:0.34)		0.69 (0.68:0.7)		0.49 (0.48:0.5)	
Gender	Woman	0.43 (0.4:0.46)	*0.00*	0.9 (0.87:0.94)	*0.00*	0.64 (0.62:0.66)	0.09	0.24 (0.21:0.26)	*0.00*	0.64 (0.6:0.68)	*0.02*	0.42 (0.4:0.44)	*0.00*
	Man	0.55 (0.53:0.57)		0.82 (0.8:0.83)		0.67 (0.66:0.68)		0.34 (0.33:0.34)		0.68 (0.67:0.69)		0.49 (0.48:0.5)	
Vint, Jeff	Injected	0.53 (0.49:0.57)	0.69	0.79 (0.76:0.83)	0.06	0.65 (0.62:0.68)	0.11	0.3 (0.28:0.32)	*0.00*	0.65 (0.63:0.67)	*0.00*	0.46 (0.44:0.47)	*0.00*
	Have not injected	0.54 (0.52:0.56)		0.83 (0.82:0.85)		0.67 (0.66:0.68)		0.33 (0.32:0.34)		0.69 (0.67:0.7)		0.49 (0.48:0.5)	
Heroin, Siretsi	Injected	0.55 (0.52:0.58)	0.17	0.89 (0.86:0.91)	*0.00*	0.7 (0.69:0.72)	*0.00*	0.35 (0.34:0.36)	*0.00*	0.7 (0.69:0.72)	*0.00*	0.51 (0.5:0.51)	*0.00*
	Have not injected	0.53 (0.5:0.55)		0.77 (0.76:0.79)		0.64 (0.63:0.65)		0.3 (0.29:0.32)		0.65 (0.63:0.66)		0.46 (0.45:0.47)	
Desomorphine	Injected	0.67 (0.64:0.7)	*0.00*	0.73 (0.71:0.76)	*0.00*	0.7 (0.68:0.71)	*0.00*	0.39 (0.37:0.41)	*0.00*	0.67 (0.65:0.7)	0.82	0.52 (0.5:0.54)	*0.00*
	Have not injected	0.49 (0.47:0.5)		0.86 (0.84:0.88)		0.65 (0.64:0.67)		0.31 (0.3:0.32)		0.68 (0.67:0.69)		0.48 (0.47:0.48)	
Suboxone/Subutex	Injected	0.43 (0.4:0.46)	*0.00*	0.8 (0.77:0.82)	*0.00*	0.6 (0.58:0.61)	0.04	0.32 (0.3:0.33)	0.24	0.65 (0.63:0.67)	*0.00*	0.47 (0.45:0.48)	*0.00*
	Have not injected	0.57 (0.55:0.59)		0.83 (0.82:0.85)		0.69 (0.68:0.7)		0.33 (0.32:0.34)		0.69 (0.68:0.7)		0.49 (0.48:0.5)	
Condom use during the last 6 months^α^	Did not have sex during the last 6 months	0.48 (0.45:0.51)	*0.00*	0.75 (0.72:0.77)	*0.00*	0.6 (0.58:0.62)	*0.00*	0.29 (0.27:0.3)	*0.00*	0.59 (0.56:0.61)	*0.00*	0.42 (0.41:0.44)	*0.00*
	Always/mostly	0.56 (0.54:0.58)		0.88 (0.86:0.9)		0.7 (0.69:0.72)		0.36 (0.35:0.37)		0.72 (0.71:0.73)		0.52 (0.51:0.53)	
	Sometimes/never	0.59 (0.55:0.64)		0.77 (0.73:0.81)		0.67 (0.65:0.7)		0.3 (0.28:0.32)		0.72 (0.7:0.75)		0.49 (0.48:0.51)	
HIV knowledge	Incorrect	0.57 (0.53:0.6)	0.05	0.85 (0.82:0.87)	0.13	0.69 (0.67:0.71)	0.01	0.3 (0.29:0.31)	*0.00*	0.69 (0.68:0.71)	*0.00*	0.48 (0.47:0.49)	0.18
	Correct	0.53 (0.51:0.55)		0.82 (0.8:0.84)		0.66 (0.65:0.67)		0.35 (0.34:0.36)		0.66 (0.65:0.68)		0.49 (0.48:0.5)	
HIV testing and know result	Never tested	0.77 (0.73:0.8)	*0.00*	0.52 (0.51:0.54)	*0.00*	0.66 (0.64:0.67)	0.11	0.32 (0.31:0.33)	*0.00*	0.58 (0.57:0.59)	*0.00*	0.44 (0.43:0.45)	*0.00*
	Yes	0.48 (0.46:0.5)		0.9 (0.89:0.92)		0.67 (0.66:0.68)		0.35 (0.33:0.36)		0.91 (0.89:0.92)		0.6 (0.59:0.61)	
	No	0.32 (0.22:0.42)		0.94 (0.78:1.1)		0.6 (0.5:0.7)		0.25 (0.21:0.3)		0.79 (0.72:0.86)		0.49 (0.45:0.53)	

^α^“no response” are missing in the analysis

There were large, statistically significant differences between usage rates of different types of drugs in the NSP and PDI groups, with the PDI group having higher rates of use of vint/jeff (24.4 v. 17.0, *p* value = 0.00), heroin (53.1 v. 45.4, *p* value = 0.00), Suboxone/Subutex (29.2 v. 24.7, *p* value = 0.00), and tropicamid/ketamine/calypso (9.1 v. 7.8, *p* value = 0.00). In the PDI group, users of vint/jeff had lower drug, sex, and overall risk, compared to those who had not used vint/jeff, all of which were statistically significant. Results were statistically insignificant in the NSP group. Users of heroin had higher risk in all categories in both NSP and PDI groups, all statistically significant except for drug risk in the NSP group (0.55 v. 0.53, *p* value = 0.17). Users of desomorphine had a mixed picture, but overall risk was higher in those who used in both the NSP (0.7 v. 0.65, *p* value = 0.00) and PDI groups (0.52 v. 0.48, *p* value = 0.00).

Regular condom use was higher in the NSP sample than the PDI sample (27.7% compared to 17.7%, *p* value = 0.00). In the NSP and PDI samples, risk of all types declined with condom use, and this was statistically significant in all cases, in those who had had sex in the 6 months prior to survey. This is possibly related to interaction with harm reduction services, which provide education around disease transmission risks, including sex risk.

HIV knowledge was strikingly different between the two groups; the NSP group, who had previous documented education, had a 75.2% correct answer rate when assessed, compared to a 53.0% in the PDI group (*p* value = 0.00). HIV knowledge had a variable association with risk; those in the PDI group with more knowledge had more drug risk (0.35 v. 0.3, *p* value = 0.00) but less sex risk (0.66 v. 0.69, *p* value = 0.00). PDI risk overall did not show a statistically significant difference with different levels of HIV knowledge. Again, it is likely that the differences between the NSP group and PDI groups in terms of HIV knowledge relate to a degree to interaction with harm reduction services which provide HIV information and risk knowledge.

The number of NSP clients who had not been tested for HIV was much lower than the PDI sample, with only 20.4% never tested, compared to 70.2% in the PDI sample (*p* value = 0.00), which is unsurprising as NSP clients are offered testing during harm reduction interactions. Among those never tested for HIV, the NSP clients had the greatest risk behaviours in terms of drugs risk, and PDI clients never tested had the greatest risk in sex risk. Aside from overall risk in the NSP group, all other differences in testing status and risk were statistically significant. Interestingly, overall risk was lower in the PDI sample that had been tested for HIV than in the NSP sample, possibly related to personalised fear of infection or increased degree of health knowledge and health seeking. In the PDI group, risk was highest in all categories in people who had been tested for HIV, but the same was not true for the NSP sample. It is unknown why this phenomenon exists.

A deeper analysis of sharing of syringes and other injecting equipment had shown that NSP clients practised more sharing of injecting equipment, such as cooker cup, spoon, filter, and other, than of sharing the syringe itself; 62.4% never shared syringes and 74.0% never shared cotton during the previous 6 months, compared to 41.4% who never shared equipment. Needle sharing refers equally to the all questions: “How often have you used a syringe after someone?” “How often have others used after you?” and “How often did you share syringe with others?” Over half (58.6%) of NSP participants reported that they shared injecting equipment during the previous 6 months. The situation is a bit different in PDI sample with 63.6% stating that they had shared injecting equipment in the previous 6 months and, in general, showing more risky sharing behaviours, all of which were statistically significant between the NSP and PDI group, and could relate to education received during harm reduction interaction and availability of free, sterile supplies of injecting equipment.

According to reported sources of sterile/clean syringes, we found that NSP clients mostly use syringes from the needle and syringe program (81.0%) during the previous 6 months. Other important sources for clean syringes for NSP participants were pharmacies (36.2%) and shooting galleries/places where drug users are gathered for injecting drugs (25.9%). Clean needle sources for PDI clients, as expected, were mostly pharmacies (90.0%) and shooting galleries (32.6%); only 3.6% were received from PWIDs from NSP. In Georgia, shooting galleries are usually in designated private residences that are hidden from law enforcement services and usually are coordinated with the NSP as to allow a clean supply of syringes to the gallery.

Analysis of the type of drugs used during last month showed that in comparison with PDI participants, NSP participants used more home-made drugs, such as desomorphine/“Crocodile” (27.7% v. 16.1%, *p* value = 0.00), street methadone (7.1% v. 6.9, *p* value = 0.00), and antihistamines in mixture (5.6% v. 4.0%, *p* value = 0.00).

## Discussion

In the large sample of PWIDs recruited from NSP and PDI, we found similarities in injection-related risk behaviour, while the sexual risk behaviour was different. These differences appear to be driven by the gender of PDI participants, as shown through the large deviation in risk scores across gender in the NSP and PDI group in Risk Assessment Battery. These findings suggest that PDI-type interventions may benefit from the incorporation of interventions directed to safe sex practice promotion, and that potentially, NSP participants could benefit more than PDI participants in further education.

PDI recruited young injectors and PWIDs with poor HIV testing practice, as shown by the large proportion of young PWIDs recruited by PDI compared to NSP and their lack of HIV knowledge during knowledge testing, the differences between which were statistically significant. Successful PDI in other locations has been shown to be effective in recruiting young PWIDs, who were much less likely to have ever been tested for HIV [12]. Other studies have analysed the efficacy of PDIs, including in the context of Russia, and have found them to be able to reduce injection frequency, reducing sharing of syringes and equipment, and reduce rates of unprotected sex [9]. The effect that recruitment of previously unknown, high-risk individuals could have on the PWID community is noteworthy and its successes in recruiting hidden populations, including in our context, is cause for wider evidence gathering and implementation.

PDI was successful in finding PWIDs who, for whatever reason, were not known to harm reduction services, even though such services had been established for years. Similar successes with previously under-represented populations have been documented with other PDIs, such as in Ukraine, where PDI managed to attract more young and female PWIDs who had not previously used a harm reduction service [12]. Many potential reasons have been cited as barriers to access to harm reduction activities and disease treatment in other locations, including lengthy waiting times [13], expense of private treatment [13], work schedules [14], lack of social marketing [14], unawareness of services [5], previously negative test results creating complacency [15], lack of female-oriented services [16], and even just stagnation of service efficacy due to its age [12]. It is unsure why these populations had remained unknown to harm reduction services in our sample, and acknowledgement of these previous findings and the limitation of efficacy that they can produce should be further investigated in the context of individual harm minimisation programs, such as in Georgia.

According to our study’s findings, participants of NSP were engaged in lower levels of syringe sharing, had comparatively better knowledge on HIV, and had higher HIV testing uptake, all of which were statistically significant differences between NSP and PDI groups. The link between clean needle programs and reduction in drug risk has been noted in other studies [17]. This is a welcome finding that underscores the benefits of participation in NSP, as lower levels of testing and education confer greater risk to PWIDs, their partners, families, and drug using network, through exposure, while also increasing the chance of non-testing by lack of awareness of HIV transmission risks [13]. Greater utilisation of testing also produces increased opportunities for education, counselling, and case management, with testing being the first step in management of HIV-infected individuals [11].

The rates of sharing of equipment in the studied PWID sample are a cause for concern, investigation, and education, as equipment sharing has been linked to HIV transmission independently of syringe sharing [18]. HIV transmission education needs to incorporate communication of the risk posed by all types of equipment sharing, not just syringe. This is pertinent in our sample, as we showed different rates between sharing of syringe and equipment, with sharing of equipment being more prevalent. Whether this relates to a gap in knowledge of HIV transmission, or just an artefact of drug culture, remains unknown.

NSP participants used more home-made drugs than PDI clients, with statistically significant differences, which can be considered as one of the important factors for more risky behaviour. This drift away from traditional opiates, such as heroin, to home-made and stimulant drugs has been noted by other studies, including ex-Soviet countries [12], and has promoted the need for adaptive programs to capture new and different PWIDs still at risk of HIV acquisition, but not part of the traditional drug scene [12]. Program changes need to be aware of the ever-changing drug landscape and incorporate the needs of a range of PWIDs into their implementation for holistic catchment of those at risk of HIV acquisition.

It was interesting to reveal that NSP clients did not always use syringes from the needle and syringe program. This presents several risks, such as equipment risks from repeated needle use and blunting with larger bore needles [18]. It can be considered that strict drug law and policy contributes to lack of accessibility to needle and syringe program, especially punishment measures on NSP participants and harassment of NSP staff, negatively affecting clean needle distribution among PWIDs. Criminalisation of clean needle exchange and possession has been related to increased needle reuse frequency in other locations [19]. In addition, the stigma that comes with criminalisation can negatively affect prevention activities and service efficacy, as shown in Africa [20] and Sub-Saharan Africa [21]. Criminalisation and punishment is thought to not be effective in preventing rising HIV incidence [13]. Studies of Georgian PWIDs found that between 9% and 24% of surveyed PWIDs had been subject to an administrative sentence due to drug use and between 1.2% and 5.5% of the subjects had been imprisoned [5]. Global efforts have highlighted the dual risk of high rates of injecting drug use and drug criminalisation, with efforts made to decrease HIV incidence around injecting drug use with assistance from criminal policy change, but so far, these have fallen much below their intended incidence reduction targets [22]. This is very relevant to the Georgian drug environment.

Social demographic characteristics, together with risky injecting and sexual behaviours, suggest that the NSP includes PWIDs that are more vulnerable and in greater need of HIV services. Other Georgian-based studies have found that lower rates of HIV testing and transmission knowledge were associated with lower levels of education and younger age [5], an association shown in other locations also [23]. Therefore, those with a potentially lower social demographic status represent a higher risk population among Georgian PWIDs. The follow-on risk of this is that those with lower rates of testing were more likely to partake in sharing behaviours, and it was also shown that between 45.8% and 64.0% of PWIDs sampled erroneously considered themselves to be at “low or no risk” for HIV transmission [5]. The relationship between self-assessment of HIV risk with risk behaviour is not linear or logical [15]. This further highlights the importance of HIV transmission risk knowledge and education, particularly in higher risk and vulnerable groups, as the relationship is more complex than it would seem. Other studies have discussed the risks and figures of unknown blood-borne infections in PWIDs and qualified the risk that low levels of testing and disease status knowledge can affect risk of the PWID’s social network [24]. Unknown blood-borne infection risk has been shown to be associated with increased risk behaviour [24]. As the perception of risk, testing frequency, and social demographic status are complexly linked, programs need to ensure catchment of a variety of participants, to balance the risks that come from the complicated psychological nature of behavioural risk.

For PWIDs in the both samples, sex behaviour was more risky than drug behaviour, as sex risk scores were greater than drug Risk in both NSP and PDI groups. Risky sexual behaviour is a transmission risk in of itself, but also compounds risk in PWIDs [13]. Among Georgian PWIDs studied in other samples, HIV sexual transmission risk did not seem to be applied when having sexual contact with a non-regular partner and this result was potentially hypothesised to be related to a belief that sex with a non-regular partner is not a risk for HIV transmission [5]. It has also been noted by other studies, however, that sexual risks are harder to change in participants than drug risk [23]. This could guide further program formation with scope for increased sexual risk education, and the awareness of not only the complexity of sexual risk and risk behaviour, but also the difficulty of change could lead to more innovative and focussed program changes.

According to analyses of Sexual Risk Index, female PDI participants had less risky behaviour than male PDI participants; we found the opposite among NSP male and female participants, with female NSP clients participating in more risky sexual behaviour, which could be related to participating in paid sex. Higher rates of risky sexual behaviours in females have been found in other studies, in reference to paid sex and condom use [24, 25], and potentially inversely-related drug and sexual risks noted due to paid sex and economic independence, which could explain some of the discrepancy between male and female drug and sex risk [26]. Female PWIDs have been previously identified as higher risk for HIV infection compared to male PWIDs [16, 27], and their under-representation in these Georgian harm reduction activities indicates a population at risk of increased HIV acquisition and in need of greater targeting.

Interventions addressing the risks of sexual transmission of HIV among PWIDs and between PWIDs and their sexual partners, and onwards to other populations, are urgently required. The dual risk of injection practise as well as sex risk has been well documented, and this compound dual risk has been noted to be an important factor in HIV spread [24]. PWIDs have been found in some locations to be twice as likely to have multiple partners and practise low levels of condom use [27]. Programs tailored to PWID needs have been shown to have statistically significant positive effects on sexual risk behaviour in other locations [14]. The awareness of this should guide program implementation due to the weight of risk placed on PWID populations when dual risk is present.

Additionally, our analysis suggests that young people from the PDI sample have less Drug Index risk than older PDI people, the difference of which was statistically significant. This difference was not seen in the NSP sample with any statistical significance. Young Georgian PWIDs people have previously been identified as a target population in need of intensified harm reduction efforts, with young peer educators and school programs recommended to decrease the risk of harmful activities among this population [5]. The increased risk that young people are placed at in regards to HIV transmission has been noted in other studies [16, 22, 24, 26] and it has been hypothesised that younger people assess themselves at a comparatively lower risk related to gaps in the HIV transmission knowledge [16]. As stated, younger people have been shown in other locations to be less likely to have ever been tested for HIV [12]. Again, it is the complex interplay of age, self-risk assessment, and practise that creates a comparatively high-risk environment for young people and education, underscoring the need for broad catchment of participants, tailored and focussed harm reduction activities, and multimodal program implementation.

## Conclusion

Rates of HIV in Georgia, and surrounding region, are increasing. Despite successful harm reduction programs, some populations of PWIDs remain difficult to reach. There are differences in knowledge and risk across the drug using population, which affects the efficacy of harm reduction programs, with groups that remain unknown to harm reduction services showing significant differences from previously known populations. Study findings and recommendations can be used for better planning and execution of HIV prevention and educational programs in the country. The utility of PDI programs is highlighted in reaching previously under-represented populations and their use in other locations has been shown to provide a more resonant and motivated risk behaviour change than other forms of communication, such as mass media [23]. The results of our study provide much insight into at-risk populations, risk assessment, actual risk behaviour, HIV knowledge, and age and gender differences, providing much evidence on how the program should be guided into the future to have a stronger impact on PWIDs in Georgia. The efficacy of the PDI in recruiting previously unknown clients, with different demographic background and risk profile, illustrates the power of this sort of harm reduction activity and is useful to demonstrate the usefulness of PDI in this context, not only regionally, but also globally, and to advocate for the continued reduction in global burden of disease from HIV and blood-borne diseases. The utility of harm reduction programs is in their innovation and responsiveness to need and their ability to investigate and adapt to implementation gaps that allow populations to be placed at higher risk. By being able to target high-risk groups, as well as have broad capture of populations and have an impactful benefit on those who interact with programs, harm reduction activities have the chance to vastly improve the morbidity and mortality rates of those of concern and greatly improve general public health.

## Limitations

The cross-sectional study design does not allow us to determine causal relationships between outcomes and explanatory variables. As both RDS and consecutive sampling strategies were concurrently applied, the conclusions may not be generalisable to the entire population of PWIDs in Georgia. Even though interviews were conducted by the trained and experienced personnel working with PWIDs, sensitivity of the questions, as well as stigma surrounding HIV/AIDS and sexual practice, may result in informational bias. Further analyses and qualitative research is needed to analyse obstacles for PWIDs in receiving clean syringes and other HIV prevention services at low threshold services.

Despite these limitations, our study provides useful information on the efficacy of PDI among Georgian PWID and highlights important differences between PWID being reached by harm reduction interventions and those that are not. These findings will enhance the improvement of HIV interventions among PWID in the Republic of Georgia.

## Abbreviations

AIDS: Acquired Immune Deficiency Syndrome
DOT: Directly Observed Therapy
GEL: Georgian Lari
GFATM: Global Fund for AIDS, Tuberculosis and Malaria
GHRN: The Georgian Harm Reduction Network
HBV: Hepatitis B Virus
HCV: Hepatitis C Virus
HIV: Human Immunodeficiency Virus
IBBSS: Integrated Biological and Behaviour Surveillance Survey
MSM: Men who have Sex with Men
NSP: Needle-Syringe Program
PDI: Peer-Driven Intervention
PWID: People Who Inject Drugs
RAB: Risk Assessment Battery
RDS: Respondent-Driven Sampling
VCT: Voluntary Testing and Counselling
WHO: World Health Organisation
